# Hints in Gynecological Practice

**Published:** 1891-02

**Authors:** M. Yarnall

**Affiliations:** 1501 Washington Avenue; St. Louis


### HINTS IN GYNECOLOGICAL PRACTICE.

BY M. YARNALL, M. D., ST. LOUIS.

In all great communities the practice of medicine drifts towards specialties.

Practitioners obtaining a reputation in any particular department or line of practice confine themselves, sooner or later, to it; unfortunately the specialist in many instances devotes himself to local treatment, forgetting the general condition; particularly is this true of those educated as specialists.

On the other hand, the general practitioner, on undertaking a specialty, often ignores, to a greater or less degree, topical treatment.

However, at the present time a broader view is gradually being manifested, and the general as well as the local condition is being considered.

Perhaps in no department has more controversy been found than in gynecology.

It is probable that menstruation and its irregularities will never be controlled to the same extent as are the bowels and their functions, and the difficulty in finding drugs that would influence the uterine system in a like manner has induced practitioners to ignore general treatment, which in the majority of cases is more essential than local.

In a system of organs where a multitude of functional activities are so delicately adjusted as in the case of the female generative system, a destruction of equilibrium may be determined by a variety of exciting causes; the exalted nervous sensibilities in females of the more enlightened nations have tended greatly to develope the neuræsthenia of uterine diseases.

A large proportion of these reflex disorders occurs during or immediately after puberty, and may exhibit peculiarities indicating serious systemic disturbances, and some are so intimately associated with these organs as to be cured by remedies directed to them, both general and local. Insomnia, headache, neuralgia, hysteria in its various forms, and numerous other ailments, have been repeatedly shown to have their origin in the sexual organs.

The pathological conditions of morbid uterus modify the process of organic life, and through the sympathetic nervous system by direct or reflex action control, in a manner, the process of assimilation.

There can be no question that inanition is a most important cause of uterine disease, and we usually observe associated with, and often caused by, this chronic starvation, general condition of neurasthenia and debility. With the debility comes an undue softening of all the muscles of the body, including the uterus; and with the neurasthenia there are aches and pains in all the important nerve centers. Whether we call it a want of nervous tone, inanition, or undue softness, it makes but slight difference; the question is, what treatment shall be adopted?

Constipation is almost universally present in women, and it deserves special consideration in treating all uterine disorders; the nervous system must be cared for, and bad habits of whatever nature corrected as far as possible.

Pain being the prominent symptom in most uterine troubles, and remedies being at hand and reliable, the indications are clear and the treatment simple. In execution, however, it is not a simple problem; immediate relief is not alone to be considered. If opiates be resorted to for frequently recurring pain, a habit will soon be formed that is no less a calamity than the disease. While, therefore, opium and its preparations are reliable remedies, and in many cases indispensable, they should be administered as seldom and as sparingly as possible.

In all cases of uterine trouble the necessity for a thorough examination is apparent. By touch, single or bimanual, by the speculum, and by the uterine sound, the condition of all the pelvic organs should be investigated. Such an examination is often as valuable for its negative as for its positive results, and no practitioner fulfills his duty to his patient or is just to himself who treats any case of uterine trouble for any length of time without making such examination.

But the principal treatment in the majority of uterine cases is the tonic treatment. They may be hæmatic, stomachic and nervous—either or all. Iron stands at the head of the list. It is not only an hæmatic, and in proper conditions a promoter of digestion, but it decidedly augments pelvic congestion, and therefore has an emmenagogue effect. The forms at command are so numerous as to meet the requirements of any case, yet the old fashioned muriated tincture of iron is still perhaps unsurpassed when borne without discomfort. With iron may be combined nux vomica or strychnia and quinine, and in our country, in a large section of which malaria is such a constantly acting depressant, the latter may be given with a free hand.

Uterine disturbances, for the sake of distinction, are divided into different classes, yet they, to a great extent, are dependent upon the same cause, uterine inertia.

Amenorrhœa, except in cases of congenital atresia, dysmenorrhœa, both congestive, neuralgic and membranous, although the obstructive form may require surgical interference.

Menorrhagia, where it is not dependent upon other than uterine troubles, displacements, polypi, and fibrous growths; and abortions, unless due to some specific taint, each and all may be greatly benefited by a judicious tonic treatment.

Viburnum prunifolium has long been noted for its beneficen effect on the uterine system. With this as a basis, the combination entitled Dioviburnia has earned for itself an enviable reputation. With it may be combined any of the remedial agents calculated to meet special indications. As a uterine tonic it is in itself unequaled. It not only aids digestion and strengthens the general system, but directs its action especially to the uterus and its appendages, restoring the relaxed condition that superinduces leucorrhœa, displacements, etc., and mollifying the aches and pains, and the various and complicated nervous manifestations that are so distressing in all cases of uterine troubles.

Too much cannot be said of the judicious use of electricity, which is applicable to such a variety of disorders.

It is scarcely in the province of this paper to enter into details; it may be said, however, that its use is beneficial in connection with almost any rational course of treatment that may be adopted.

1501 Washington Avenue.

